# Influence of tow duration on catch performance of trawl survey in the Mediterranean Sea

**DOI:** 10.1371/journal.pone.0191662

**Published:** 2018-01-22

**Authors:** Antonello Sala

**Affiliations:** Italian National Research Council (CNR), Institute of Marine Sciences (ISMAR), Ancona, Italy; Aristotle University of Thessaloniki, GREECE

## Abstract

The aim of this study was to assess the effect of tow duration on catch per unit of swept area (*CPUE*), trawl catch performance, and the proportion of the species caught in a trawl survey. Longer tows are expected to have a greater probability of catching species. An average of 26 species were caught in the first 30 minutes, whereas only about one additional species was caught in the next 30 minutes in longer tows. The shorter tows involved a decrement in catch weight for 11 of the 12 target species sampled, demonstrating that tow duration did affect catch per unit of swept area *CPUE*. The shorter tows were associated with a significant reduction of the overall *CPUE* in terms of weight of the main target species and of the total catch (circa 60%). The same strong reduction of around 70% was found in particular for European hake (*Merluccius merluccius*) and surmullet (*Mullus* spp) and 50% for Nephrops (*Nephrops norvegicus*). The shorter tows were less efficient in catching large-sized hake, surmullet, Nephrops, Atlantic horse mackerel (*Trachurus trachurus*), and poor cod (*Trisopterus minutus*), even though the difference was significant only for Nephrops. Regardless of the *p-value* statistic, these findings suggest that the continuity of survey time series would be severely impaired by changing tow duration. Further work is required to explore a way to reduce tow duration without reducing *CPUE*.

## Introduction

In 1993, the European Commission prompted the adoption of a survey programme for the demersal resources assessment in the Mediterranean Sea. The Mediterranean international trawl survey (MEDITS) programme was started at the end of 1993 [1]. All Member countries have adopted the MEDITS protocols, which describe the sampling gear, the survey design and basic data analysis that should be applied [1]. Since 2012, the programme involves the study of a main list of 84 reference species, including fish, molluscs, and crustaceans [1]. The protocol envisages a tow duration of 30 minutes for depths less than 200 m or of 60 minutes for depths greater than 200 m. This provision aims to reduce the influence of longer net setting time in the tows performed at greater depths. Some studies [2] have suggested that a smaller sampling unit could increase the accuracy of density estimates in marine surveys. In addition, reduced tow duration provides other benefits, such as savings in survey time and operating expenses and less opportunity for underwater obstacles to damage the gear or to cause a tow to be aborted.

Experiments in other areas [3–5] have investigated whether catch amount is proportional to tow duration, in particular whether larger fish, which can swim in front of the net for a long time, are undersampled in short tows. Godø et al. [3] and Walsh [5] found that the catch amounts obtained from shorter and longer tows were not significantly different. These data were unexpected, because given the greater swimming endurance that comes with larger size [6,7] a shorter tow duration should have produced lower *CPUE* and mean size. Godø et al. [3] suggested two mechanisms to explain their findings: an underestimation of the effective swept area or a time-varying avoidance behaviour of fish (“catch-by-surprise”). Since both mechanisms involve an increase in catch amount that is not dependent on tow duration, they exert stronger effects on short tows.

The present paper describes an experiment that was conducted to assess the effect of tow duration on catch per unit of swept area (*CPUE*), trawl catch performance, and the proportion of the species caught in a trawl survey. This study was carried out to seek information on the issue whether tow duration could be reduced under the aim to avoid sub-sampling as well as to increase the total number of stations which improves the accuracy of survey estimates.

## Material and methods

### Ethics statement

All sea trials and fishing practices on board the Italian research vessel “S. Lo Bianco” have been conducted in accordance with the experimental fishing permit DG PEMAC 0007137, granted by the Italian Ministry of Agriculture and Forestry (Fishery and Aquaculture directorate). No other authorization or ethics board approval was required to conduct the study. This study did not involve endangered or protected species. Trawl catches were used just for research purposes and information on animal welfare and steps to ameliorate suffering and methods of sacrifice is not applicable, since the animals were not exposed to any additional stress other than that involved in commercial fishing practices.

### Fishing gear and research vessel

A trawl called GOC 73 (S1 File) was used in the present experiment, as established by the MEDITS protocol [1]. The tests were carried out on board the Italian R/V “S. Lo Bianco” (660 hp). Gear performance (i.e., door spread and horizontal and vertical net opening) was measured during each haul using a SCANMAR system. Data acquisition was automatically controlled through a laptop which checked correct system functioning in real time through customised software [8]. Measurements aimed principally to obtain detailed, real-time, gear performance data.

Tow duration was considered as the interval from the achievement of optimum gear opening to the time when speed was reduced to recover the warp. As recommended by the MEDITS survey handbook [1], all the systematic procedures to standardize tow durations have been followed in the present study.

### Sampling areas

A 3-day fishing trip was conducted in the Central Adriatic Sea with trials performed on the same fishing grounds. A single sampling area of 5x10 nautical miles off Ancona (Italy) was used for all the tows to maximise the homogeneity of the catch (abundance and species), thus avoiding other random and fixed effects. The assumption of this study was that the effect of tow duration on catch per unit of swept area (*CPUE*), trawl catch performance, and the proportion of the species caught would be the same regardless of the study area. The depth of the area was 50–70 m because this condition involves a shorter setting time of the gear (only 2–3 minutes) compared with deeper waters.

### Sampling methodology

Tow duration was 30 minutes (*T30*) or 60 minutes (*T60*). Overall, 14 valid alternate hauls, seven for each tow duration, were analysed. The MEDITS sampling methodology [1] envisages a vessel speed of 3 knots and exclusively daytime hauls. The species found in the catch were divided into three categories: a) the main MEDITS target species (G1); b) the other MEDITS target species (G2); and c) non-target species (G3). Category G1 includes 41 species, 9 demersal (3 fish, 4 crustaceans, 2 cephalopods) and 32 Selachians. The list of reference species is reported in the MEDITS survey handbook [1]. The MEDITS protocol was applied to the collection of biological data, except that the individuals of all species were counted and weighed, irrespective of their category.

### *CPUE* data processing

Only the species that were caught regularly and abundantly were included in the analysis. This led to the exclusion of some G1 and G2 reference species: broadtail shortfin squid *(Illex coindetii)*, caramote prawn *(Melicertus kerathurus)*, common smooth-hound *(Mustelus mustelus)*, axillary seabream *(Pagellus acarne)*, common pandora *(P*. *erythrinus)*, greater forkbeard *(Phycis blennoides)*, common cuttlefish *(Sepia officinalis)*, picarel *(Spicara smaris)*, and spiny dogfish *(Squalus acanthias)*.

For each haul *i*, the numbers of individuals, the weight of each species and the *Richness r* (number of species caught per tow) were normalized by the swept area *SWA*[km^2^], which was calculated based on the mean horizontal net opening HNO[m] (as measured by the SCANMAR system), vessel speed TS[kn] (as measured by the Doppler log), and tow duration TT[min]:

$$SWA_{i}=TT_{i}\times HNO_{i}\times TS_{i}\times \frac{60\times 1852}{3600\times 10^{6}}$$ (1)

According to McConnaughey and Conquest [9], as the mean and the confidence intervals (CI) of the original (non-transformed) data may be oversensitive to extreme values, the Geometric Mean was used as an estimator of the *Richness* and *CPUE* in terms of number of individuals (*CPUE*^*n*^) and weight (*CPUE*^*w*^) of each species *s* and for each tow duration *t* (*T30* and *T60*). It was computed by exponentiating the mean of the *log*-transformed data (*r*/*NEA*, *n/SWA* and *w/SWA*) and subtracting one, and used for all the comparisons [9,10]:

$$\begin{array}{ll} CPUE_{\left(s,t\right)}^{n}=exp\left(\frac{\sum_{i}{log}_{e}\left(\frac{n_{\left(i,s,t\right)}}{SWA_{i}}+1\right)}{N_{t}}\right)-1 & CPUE_{\left(s,t\right)}^{w}=exp\left(\frac{\sum_{i}{log}_{e}\left(\frac{w_{\left(i,s,t\right)}}{SWA_{i}}+1\right)}{N_{t}}\right)-1\\ r_{\left(t\right)}^{}=exp\left(\frac{\sum_{i}{log}_{e}\left(\frac{r_{\left(i,t\right)}}{SWA_{i}}+1\right)}{N_{t}}\right)-1 & \end{array}$$ (2)

where *N*_*t*_ is the total number of hauls for each tow duration.

As described in detail by Fiorentini et al. [11] and Dremière et al. [12], the mean *CPUE* and *Richness* values were compared by applying Student’s *t*-test to the *log*-transformed data in order guarantee the normality and homogeneity of variances.

Following the procedures outlined in Finney [10], an *Efficiency coefficient*, in terms of absolute difference and ratio (%) between tow duration *T30* and *T60* (*E*), was computed for the number of individuals (No/km^2^) and weight (kg/km^2^) for each species *s*, and for the difference in *Richness*. Based on Eq (2) the coefficients could be obtained as follows:

$$\begin{array}{l} E_{\left(s\right)}^{n}=CPUE_{\left(s,t=T30\right)}^{n}-CPUE_{\left(s,t=T60\right)}^{n}\\ E_{\left(s\right)}^{w}=CPUE_{\left(s,t=T30\right)}^{w}-CPUE_{\left(s,t=T60\right)}^{w}\\ E_{}^{r}=CPUE_{\left(t=T30\right)}^{n}-CPUE_{\left(t=T60\right)}^{n} \end{array}$$ (3)

$$\begin{array}{l} E{\%}_{\left(s\right)}^{n}=exp\left(\frac{\sum_{i}{log}_{e}\left(\frac{n_{\left(i,s,t=T30\right)}}{SWA_{i}}+1\right)}{N_{t}}-\frac{\sum_{i}{log}_{e}\left(\frac{n_{\left(i,s,t=T60\right)}}{SWA_{i}}+1\right)}{N_{t}}\right)\\ E{\%}_{\left(s\right)}^{w}=exp\left(\frac{\sum_{i}{log}_{e}\left(\frac{w_{\left(i,s,t=T30\right)}}{SWA_{i}}+1\right)}{N_{t}}-\frac{\sum_{i}{log}_{e}\left(\frac{w_{\left(i,s,t=T60\right)}}{SWA_{i}}+1\right)}{N_{t}}\right)\\ E{\%}_{}^{r}=exp\left(\frac{\sum_{i}{log}_{e}\left(\frac{r_{\left(i,t=T30\right)}}{SWA_{i}}+1\right)}{N_{t}}-\frac{\sum_{i}{log}_{e}\left(\frac{r_{\left(i,t=T60\right)}}{SWA_{i}}+1\right)}{N_{t}}\right) \end{array}$$ (4)

### Catch comparison rate by size

The catch (*CPUE*) rate by size of *T30* and *T60* tows was compared using a method that was devised by Krag et al. [13] modified for this study to overcome the requirement of paired experimental data; this allowed using the unpaired experimental data collected during alternate hauls of the experiment. The modified analysis was conducted separately for each main species as described below. Besides tow duration, catch efficiency may also be affected by haul site and by the number and size of the individuals of the reference species that are available at the time of the haul.

The catch comparison methodology suggests that specimen number and size should have been on average roughly the same for all tows. This assumption was applied as a measure to interpret the catch comparison rate by size pooled over hauls for *T30* and *T60* tows, because the data used for the comparisons were not collected in pairs. The summed catch comparison rate by size, *cc*_*ℓ*_, where *ℓ* stands for body length (for fish) and carapace length (for Nephrops), was calculated as follows:

ccℓ=∑i=1Nnℓikratioi∑i=1Nnℓikratioi+∑j=1Mmℓjkratioj (5)

where *n*_*ℓi*_ are the number of specimens of each length class *ℓ* caught in *T30* tow *i*, and *m*_*ℓj*_ the number caught in *T60* tow *j*. *N* and *M* are the total number of hauls conducted respectively with tow type *T30* and *T60*. If the catch efficiency of *T30* and *T60* tows and the number of hauls are equal (*N* = *M* as in the present case), then the expected value of the summed catch comparison rate is 0.5. In the case of a different number of hauls (*N≠M*), the baseline value indicating no difference in catch performance between the tow types would be *N/(N+M)*. Finally, terms *kratios* are the ratio of the swept area to its maximum value attained in the sea trials (see *kratio* in S1 Table), for tow *i* and *j*, respectively.

The experimental *cc*_*ℓ*_ is often modelled by the function *cc*(*ℓ*, ***ν***) [13,14], which has the following form:

$$cc(\ell ,\nu )=\frac{exp\left(f\left(\ell ,\nu_{0},\ldots ,\nu_{k}\right)\right)}{1+exp\left(f\left(\ell ,\nu_{0},\ldots ,\nu_{k}\right)\right)}$$ (6)

where *f* is a polynomial of order *k* with coefficients *v*_*0*_ to *v*_*k*_. Thus, *cc*(*ℓ*, ***ν***) is the probability of finding a specimen of length *ℓ* in the catch of one *T30* haul, since it is found in the catch of a *T30* and/or *T60* haul. The values of ***v***, describing *cc*(*ℓ*, ***ν***), are estimated by minimising Eq (7). The inner summations in Eq (7) are the sums over the hauls conducted respectively with tow type *T30* and *T60*. The outer summation in Eq (7) is the sum over length classes *ℓ*.

$$-\sum_{\ell }\{\sum_{i=1}^{N}\frac{n_{\ell i}}{q_{i}}\times ln\left(cc\left(\ell ,\nu \right)\right)+\sum_{j=1}^{M}\frac{m_{\ell j}}{q_{j}}\times ln\left(1-cc\left(\ell ,\nu \right)\right)\}$$ (7)

Minimising Eq (7) is equivalent to maximising the likelihood of the data obtained from the hauls (see [15] for additional information). In Eq (6), I considered an *f* of up to a 4^th^ order with parameters *v*_*0*_, *v*_*1*_, *v*_*2*_, *v*_*3*_ and *v*_*4*_. Leaving out one or more of parameters *v*_*0*_*…v*_*4*_ provided 31 additional models that were considered as potential models to describe *cc*(*ℓ*, ***ν***). Multi-model inference was applied based on these models, to describe *cc*(*ℓ*, ***ν***) according to the ability of each model, compared with the others, to fit the experimental data [16]. In the resulting model, hereinafter designated as the combined model, all the individual models were ranked and weighted according to their AICc values [16]. AICc is the AIC [17] with correction for finite sample sizes. Models yielding AICc values within +10 of the value of the model with the lowest AICc were considered to contribute to *cc*(*ℓ*, ***ν***) based on the procedure described by Katsanevakis [18]. The ability of the combined model to describe the experimental data was assessed based on its *p*-value; its eligibility as a candidate model therefore requires the *p*-value to be > 0.05 [19]. In case of poor-fit statistics (*p*-value < 0.05; deviance >> DOF), the deviations between the experimental catch comparison points and the fitted curve were examined to determine whether they were due to structural problems in describing the experimental data with the combined model or to data overdispersion.

Confidence intervals for the catch comparison curve were estimated using a double bootstrap method. The procedure accounted for the uncertainty due to between-haul variation in catch efficiency and in the availability of specimens of different sizes by selecting *N* hauls with replacement from the pool of *T30* tows, and *M* hauls with replacement from the pool of *T60* tows during each bootstrap repetition. Within-haul uncertainty in the size structure of the catch data was accounted for by randomly selecting specimens with replacement from each selected haul separately. These data were then combined as described above, and the catch comparison curve was estimated. A total of 1000 bootstrap repetitions were performed and Efron 95% CI [20] was calculated for the catch comparison curve. Incorporation of the above-described combined model approach in each bootstrap repetition accounted for any additional uncertainty in the catch comparison curve due to model selection. To determine whether the difference in catch efficiency between shorter and longer tows was significant, length classes without overlap between the 95% CI and the baseline were examined for any difference in catch performance between the two tow types.

### Catch comparison ratio by size

The catch comparison rate by size, *cc*_*ℓ*_, provides information on the average difference in catch efficiency between *T30* and *T60* tows. The relative catch efficiency by size of each tow was quantified by the catch ratio by size, *cr*_*ℓ*_, which can be directly calculated from *cc*_*ℓ*_. For the experimental data, the average catch ratio for a length class *ℓ* can be expressed as follows:

crℓ=1N∑i=1Nnℓikratioi1M∑j=1Mmℓjkratioj (8)

Simple mathematical manipulation based on Eqs (5) and (8) yields the following general relationship between catch ratio and catch comparison rate:

$$cr_{\ell }=\frac{M\times cc_{\ell }}{N\times \left(1-cc_{\ell }\right)}$$ (9)

This entails that the same relationship exists for the functional forms:

$$cr(\ell ,\nu )=\frac{M\times cc(\ell ,\nu )}{N\times \left(1-cc(\ell ,\nu )\right)}$$ (10)

An advantage of using the catch ratio as it is defined by Eqs (8) and (10) is that, unlike the catch comparison rate, it provides a direct relative value of the catch efficiency of *T30* compared with *T60* tows. Moreover, the way the catch ratio is defined by Eqs (8) and (10) provides a value that is not dependent on the number of hauls of each tow type that have been performed. Thus, if the catch efficiency of shorter and longer tows is equal, *cr*(*ℓ*, ***ν***) should always be 1.0. For example, *cr*(*ℓ*, ***ν***) = 1.25 would mean that *T30* tows catch on average 25% more specimens of length *ℓ* than *T60* tows, whereas *cr*(*ℓ*, ***ν***) = 0.75 would mean that *T30* tows catch only 75% of specimens of length *ℓ* compared with *T60* tows. The CI for the catch ratio were estimated using Eq (10) and incorporating the calculation of *cr*(*ℓ*, ***ν***) for each relevant length class into the double bootstrap procedure described for the catch comparison rate. Catch ratio analysis was then used to estimate the length-dependent effect of a change from *T60* to *T30* tows on catch efficiency.

The analytical sequence described above was implemented in SELNET software [15], which was used for all analyses. SELNET has previously been applied to analyse size selectivity [21–27] and catch comparison [13, 14] data collected with trawls. This is the first time it is used to analyse the influence of tow duration on gear catch data from trawl surveys.

## Results

### *CPUE* and *Richness*

A total of 14 hauls, seven for each tow duration, were performed in the same fishing ground in reciprocal directions. Their details are reported in S1 Table. The species that were not found in more than 30% of the hauls were excluded from the analyses. Overall, 12 MEDITS target species were included, 4 of G1 category: European squid (*Loligo vulgaris*), European hake (*M*. *merluccius*), surmullet (*Mullus* spp), Norway lobster (*N*. *norvegicus*), and 8 of G2 category: bogue (*Boops boops*), musky octopus (*Eledone moschata*), European anchovy (*Engraulis encrasicolus*), sardine (*Sardina pilchardus*), Atlantic mackerel (*Scomber scomber*), common sole (*Solea solea*), Atlantic horse mackerel (*Trachurus trachurus*), and poor cod (*T*. *minutus*). According to the MEDITS survey handbook [1], the two species of *Mullus* spp, Striped red mullet (*Mullus surmuletus*) and Red mullet (*Mullus barbatus*), have been combined.

Their mean *CPUE* in terms of weight (kg/km^2^) was greater in *T60* than in *T30* tows for 11/12 species, even though the difference was significant only for Nephrops (Table 1). A greater mean *CPUE* in terms of numbers (No/km^2^) was also found for most species in *T60* tows, except for musky octopus (*E*. *moschata*) (G2), which showed a greater *CPUE* in terms of weight and numbers in *T30* tows, and poor cod (*Trisopterus minutus*), which presented a higher *CPUE* in terms of numbers in T30 tows, but lower *CPUE* in terms of weight in T60 tows (Tables 1 and 2).

**Table 1:** Geometric Mean of the *CPUE* in terms of weight (kg/km^2^).

Group	Species		*T60* [kg/km^2^]		*T30* [kg/km^2^]		*Efficiency coeff*. *(E)*			
							[kg/km^2^]	[%]	*Sig*.	
**G1**	*L*. *vulgaris*	European squid	**0.57**	*(0*.*00–2*.*97)*	**0.02**	*(0*.*00–0*.*06)*	**-0.55**	**64.8**	*0*.*274*	
	*M*. *merluccius*	European hake	**32.72**	*(23*.*12–46*.*15)*	**23.98**	*(15*.*49–36*.*86)*	**-8.74**	**74.1**	*0*.*195*	
	*Mullus spp*	Surmullets	**1.36**	*(0*.*54–2*.*60)*	**0.63**	*(0*.*00–1*.*71)*	**-0.73**	**69.0**	*0*.*196*	
	*N*. *norvegicus*	Norway lobster	**8.07**	*(5*.*81–11*.*09)*	**3.74**	*(1*.*84–6*.*91)*	**-4.33**	**52.3**	*0*.*019*	(*)
**G2**	*B*. *boops*	Bogue	**7.51**	*(1*.*03–34*.*67)*	**4.39**	*(1*.*25–11*.*90)*	**-3.11**	**63.4**	*0*.*519*	
	*E*. *moschata*	Musky octopus	**1.26**	*(0*.*00–5*.*50)*	**4.52**	*(0*.*21–24*.*14)*	**3.26**	**244.4**	*0*.*260*	
	*E*. *encrasicolus*	European anchovy	**5.59**	*(0*.*57–26*.*64)*	**2.54**	*(0*.*41–7*.*92)*	**-3.05**	**53.8**	*0*.*391*	
	*S*. *pilchardus*	Sardine	**3.91**	*(0*.*12–20*.*43)*	**2.95**	*(0*.*33–10*.*68)*	**-0.96**	**80.4**	*0*.*776*	
	*S*. *scombrus*	Atlantic mackerel	**2.45**	*(0*.*67–6*.*13)*	**0.57**	*(0*.*00–2*.*37)*	**-1.89**	**45.3**	*0*.*092*	
	*S*. *solea*	Common sole	**1.07**	*(0*.*02–3*.*21)*	**0.29**	*(0*.*00–1*.*42)*	**-0.78**	**62.4**	*0*.*246*	
	*T*. *trachurus*	Horse mackerel	**9.26**	*(3*.*15–24*.*41)*	**5.54**	*(1*.*57–15*.*65)*	**-3.73**	**63.7**	*0*.*413*	
	*T*. *minutus*	Poor cod	**1.80**	*(0*.*76–3*.*46)*	**1.05**	*(0*.*20–2*.*51)*	**-0.75**	**73.1**	*0*.*303*	
**G1**	All G1 species		**51.27**	*(36*.*07–72*.*69)*	**29.95**	*(20*.*72–43*.*10)*	**-21.32**	**59.2**	*0*.*023*	(*)
**G2**	All G2 species		**64.04**	*(36*.*17–112*.*81)*	**39.25**	*(25*.*09–61*.*11)*	**-24.79**	**61.9**	*0*.*123*	
**Total**	All species		**152.90**	*(120*.*46–194*.*00)*	**100.74**	*(73*.*06–138*.*78)*	**-52.15**	**66.1**	*0*.*025*	(*)

In parenthesis 95% confidence intervals of the G1 and G2 categories of the main MEDITS reference species for the hauls of 30 minutes or 60 minutes duration (*T30* and *T60*, respectively). An *Efficiency coefficient*, in terms of absolute difference and ratio (%) of the *CPUE* (kg/km^2^) between *T30* and *T60* (*E*), was computed to quantify the effect of tow duration. Student’s t-test for *CPUE*: (*) significant, *0*.*01<Sig*.*<0*.*05*. The Mediterranean International Trawl Survey (MEDITS) protocol establishes three species categories: a) main MEDITS target species (G1); b) other MEDITS target species (G2); and c) non-target species (G3). Category G1 includes 41 species, 9 demersal (3 fish, 4 crustaceans, 2 cephalopods) and 32 Selachians, whereas category G2 includes 42 species. The list of reference species is reported in the MEDITS survey handbook [1].

**Table 2:** Geometric Mean of the *CPUE* in terms of numbers (No/km^2^) and species *Richness r* (number of species caught per tow).

Group	Species		T60 [No/km^2^]		T30 [No/km^2^]		*Efficiency coeff*. *(E)*			
							[No/km^2^]	[%]	*Sig*.	
**G1**	*L*. *vulgaris*	European squid	**4.70**	*(0*.*00–47*.*32)*	**2.09**	*(0*.*00–18*.*02)*	**-2.61**	**54.2**	*0*.*603*	
	*M*. *merluccius*	European hake	**479.52**	*(335*.*14–685*.*90)*	**403.04**	*(269*.*49–602*.*52)*	**-76.48**	**84.1**	*0*.*445*	
	*Mullus spp*	Surmullets	**40.28**	*(17*.*13–93*.*01)*	**13.63**	*(1*.*39–88*.*52)*	**-26.66**	**35.4**	*0*.*226*	
	*N*. *norvegicus*	Norway lobster	**145.06**	*(83*.*68–250*.*92)*	**83.67**	*(32*.*94–210*.*24)*	**-61.39**	**58.0**	*0*.*234*	
**G2**	*B*. *boops*	Bogue	**85.01**	*(10*.*11–664*.*83)*	**59.22**	*(9*.*94–330*.*59)*	**-25.79**	**70.0**	*0*.*749*	
	*E*. *moschata*	Musky octopus	**3.17**	*(0*.*00–22*.*34)*	**11.31**	*(0*.*36–109*.*98)*	**8.14**	**295.1**	*0*.*362*	
	*E*. *encrasicolus*	European anchovy	**299.21**	*(87*.*96–1*,*012*.*14)*	**183.31**	*(56*.*61–588*.*68)*	**-115.90**	**61.4**	*0*.*492*	
	*S*. *pilchardus*	Sardine	**60.63**	*(5*.*12–619*.*60)*	**48.54**	*(6*.*07–346*.*06)*	**-12.09**	**80.4**	*0*.*863*	
	*S*. *scombrus*	Atlantic mackerel	**33.54**	*(5*.*04–196*.*34)*	**3.78**	*(0*.*00–57*.*25)*	**-29.75**	**13.8**	*0*.*138*	
	*S*. *solea*	Common sole	**3.77**	*(0*.*21–17*.*84)*	**0.57**	*(0*.*00–3*.*75)*	**-3.20**	**32.9**	*0*.*149*	
	*T*. *trachurus*	Horse mackerel	**664.11**	*(180*.*79–2432*.*39)*	**402.99**	*(88*.*58–1820*.*85)*	**-261.12**	**60.7**	*0*.*551*	
	*T*. *minutus*	Poor cod	**196.62**	*(97*.*64–394*.*93)*	**259.07**	*(154*.*10–435*.*09)*	**62.46**	**131.6**	*0*.*453*	
***r***	**G1**		**3.97**	*(3*.*46–4*.*54)*	**3.09**	*(2*.*49–3*.*80)*	**-0.88**	**82.3**	*0*.*030*	(*)
	**G2**		**7.51**	*(6*.*53–8*.*61)*	**7.51**	*(6*.*53–8*.*61)*	**0.00**	**100.0**	*1*.*000*	
	**All Species**		**27.16**	*(24*.*66–29*.*92)*	**26.02**	*(23*.*59–28*.*69)*	**-1.14**	**95.9**	*0*.*460*	

In parenthesis 95% confidence intervals of the main G1 and G2 MEDITS reference species for the hauls of 30 minutes or 60 minutes duration (*T30* and *T60*, respectively). An *Efficiency coefficient*, in terms of absolute difference and ratio (%) of the *CPUE* (No/km^2^) and *Richness* between *T30* and *T60* (*E*), was computed to quantify the effect of tow duration. Student’s t-test for *CPUE* and *Richness*: (*) significant, *0*.*01<Sig*.*<0*.*05*. The Mediterranean International Trawl Survey (MEDITS) protocol establishes three species categories: a) main MEDITS target species (G1); b) other MEDITS target species (G2); and c) non-target species (G3). Category G1 includes 41 species, 9 demersal (3 fish, 4 crustaceans, 2 cephalopods) and 32 Selachians, whereas category G2 includes 42 species. The list of reference species is reported in the MEDITS survey handbook [1].

The total *CPUE* in terms of weight of the main G1 target species was significantly higher (*p = 0*.*023*) in *T60* than *T30* tows (Table 1). The *CPUE Efficiency coefficient* was evident both in terms of absolute numbers and of rates, and was respectively ca. 21 kg and ca. 59% (Table 1). The main G2 MEDITS species also showed a higher *CPUE* in *T60* (64.04 kg/km^2^) compared with *T30* tows (39.25 kg/km^2^), but the *CPUE Efficiency coefficient*, ca. 25 kg, was not significant (*p = 0*.*123*). When the total catch was considered, the overall *CPUE* of *T30* tows was 100.74 kg/km^2^, i.e. 66.1% of the 152.90 kg/km^2^ estimated for *T60* tows (Table 1).

The mean *Richness* for G1 was 3.97 and 3.09 in *T60* and *T30* tows (Table 2), with a significant difference of 0.88 species/tow (*p = 0*.*030*). Overall, 27.16 species/haul were caught in *T60* tows compared with 26.02 species/haul in *T30* tows; also in this case, about one additional species was found in *T60* compared with *T30* tows (Table 2).

### Catch comparison rate and ratio

The findings of the present study allowed comparing the catch data for three G1 MEDITS species, European hake (*M*. *merluccius*), surmullet (*Mullus* spp), and Norway lobster (*N*. *norvegicus*), and two G2 species, Atlantic horse mackerel (*T*. *trachurus*) and poor cod (*T*. *minutus*) because they were the most abundant species in the catches of both tow durations.

**Fig 1:**
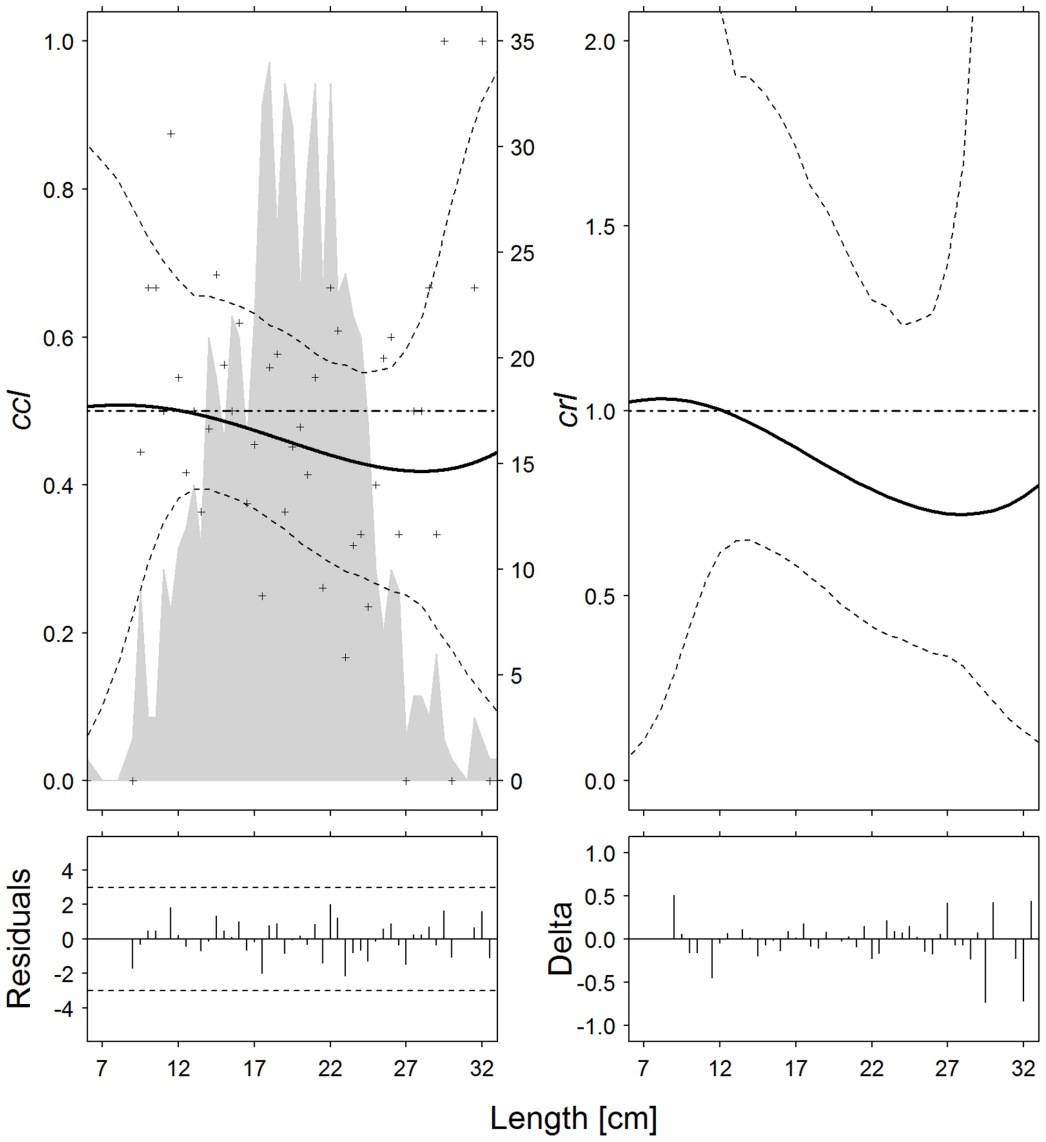
European hake (*M*. *merluccius*). Catch comparison rates (*cc*_*ℓ*_, *top left*) and catch ratio curves (*cr*_*ℓ*_, *top right*) of the 30-minute and 60-minute tows (solid curves). Crosses: experimental rates; thin dotted curves: 95% confidence intervals; grey solid curve: summed and raised catch populations for all hauls; horizontal dashed line: baseline of no effect on catch performance after changing tow duration. Partial residual plots (*bottom left*) and delta dispersion (*bottom right*) for the two models.

**Fig 2:**
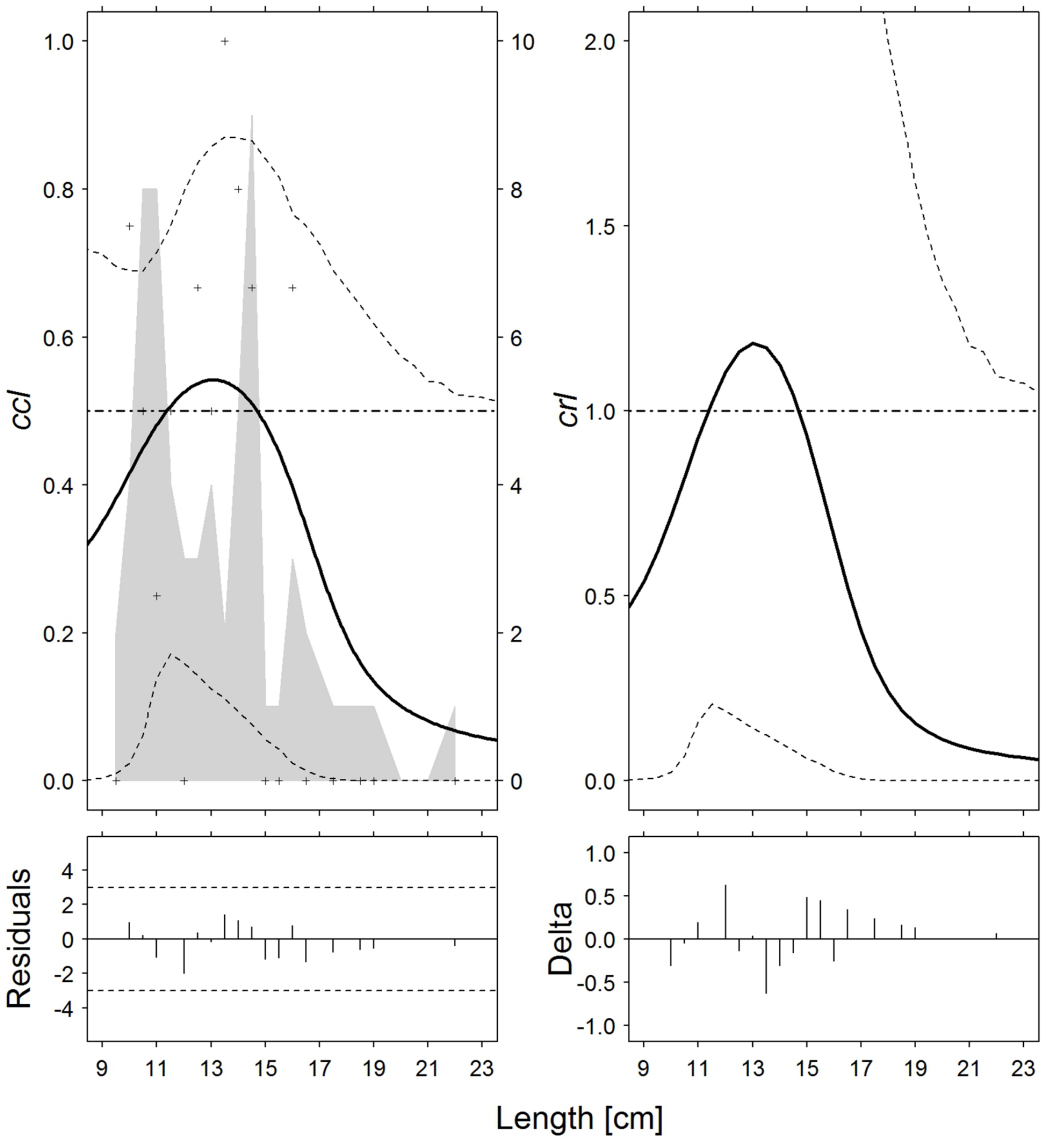
Surmullets (*Mullus* spp). Catch comparison rates (*cc*_*ℓ*_, *top left*) and catch ratio curves (*cr*_*ℓ*_, *top right*) of the 30-minute and 60-minute tows (solid curves). Crosses: experimental rates; thin dotted curves: 95% confidence intervals; grey solid curve: summed and raised catch populations for all hauls; horizontal dashed line: baseline of no effect on catch performance after changing tow duration. Partial residual plots (*bottom left*) and delta dispersion (*bottom right*) for the two models.

**Fig 3:**
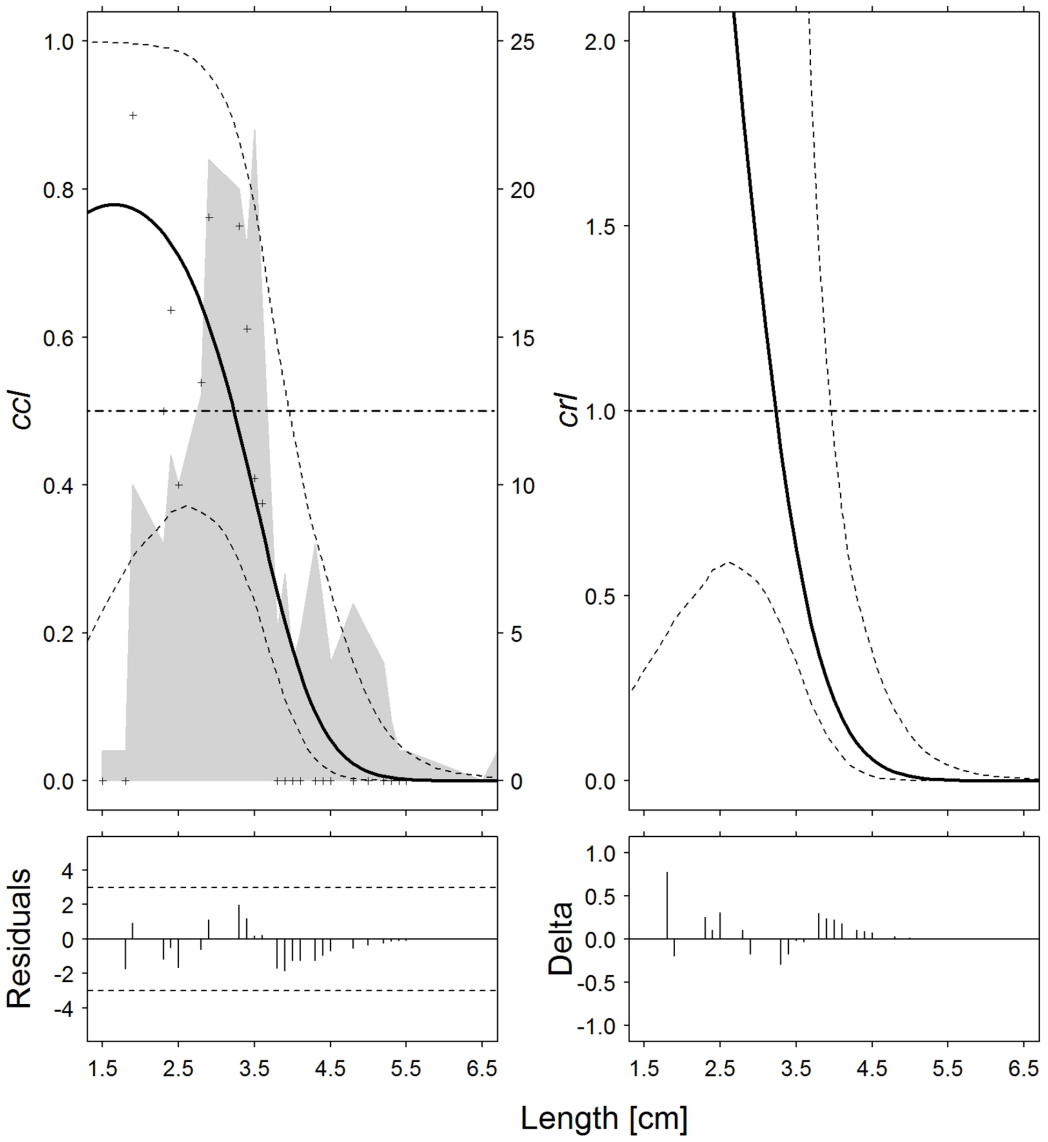
Norway lobster (*N*. *norvegicus*). Catch comparison rates (*cc*_*ℓ*_, *top left*) and catch ratio curves (*cr*_*ℓ*_, *top right*) of the 30-minute and 60-minute tows (solid curves). Crosses: experimental rates; thin dotted curves: 95% confidence intervals; grey solid curve: summed and raised catch populations for all hauls; horizontal dashed line: baseline of no effect on catch performance after changing tow duration. Partial residual plots (*bottom left*) and delta dispersion (*bottom right*) for the two models.

**Fig 4:**
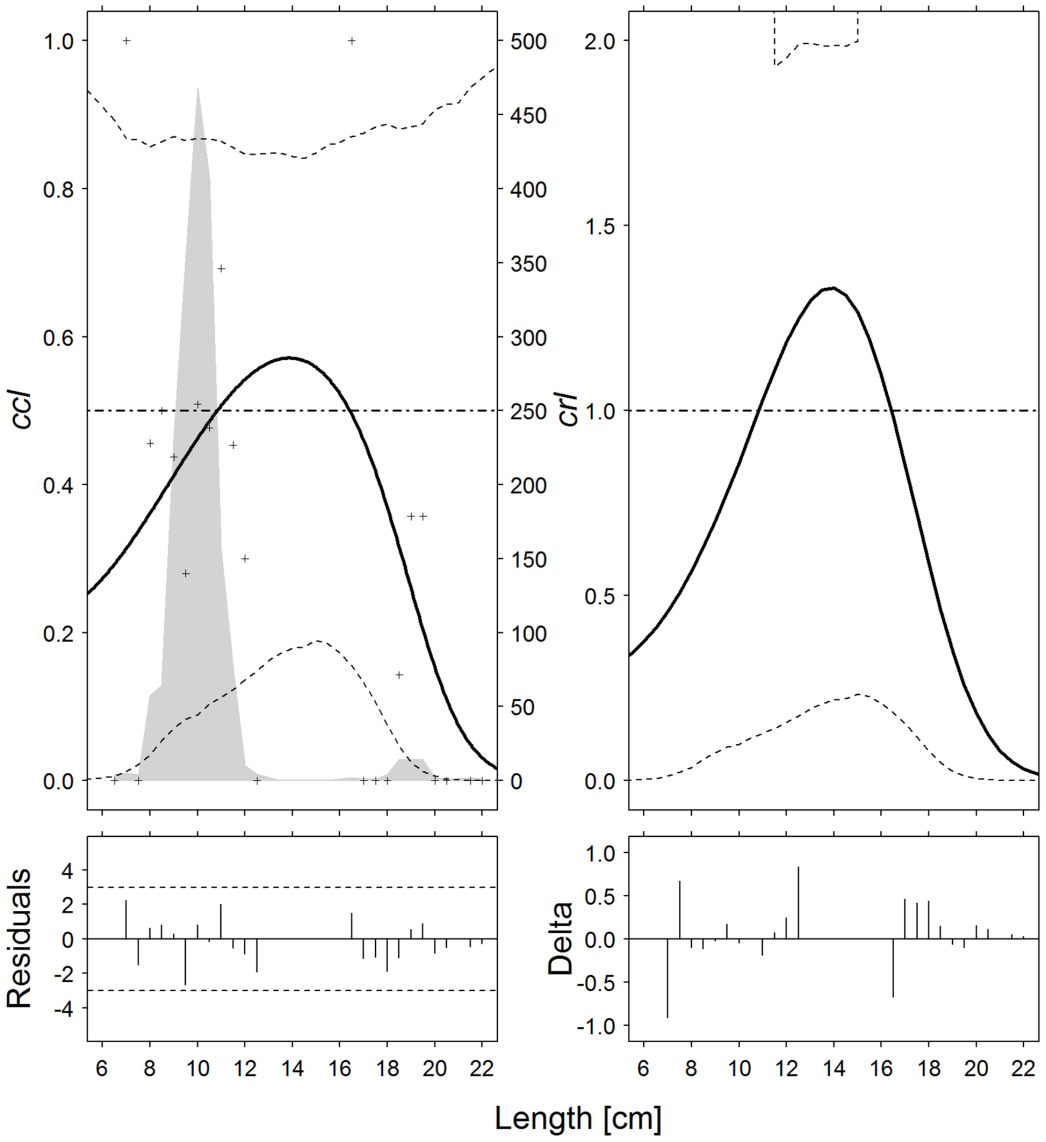
Atlantic horse mackerel (*T*. *trachurus*). Catch comparison rates (*cc*_*ℓ*_, *top left*) and catch ratio curves (*cr*_*ℓ*_, *top right*) of the 30-minute and 60-minute tows (solid curves). Crosses: experimental rates; thin dotted curves: 95% confidence intervals; grey solid curve: summed and raised catch populations for all hauls; horizontal dashed line: baseline of no effect on catch performance after changing tow duration. Partial residual plots (*bottom left*) and delta dispersion (*bottom right*) for the two models.

**Fig 5:**
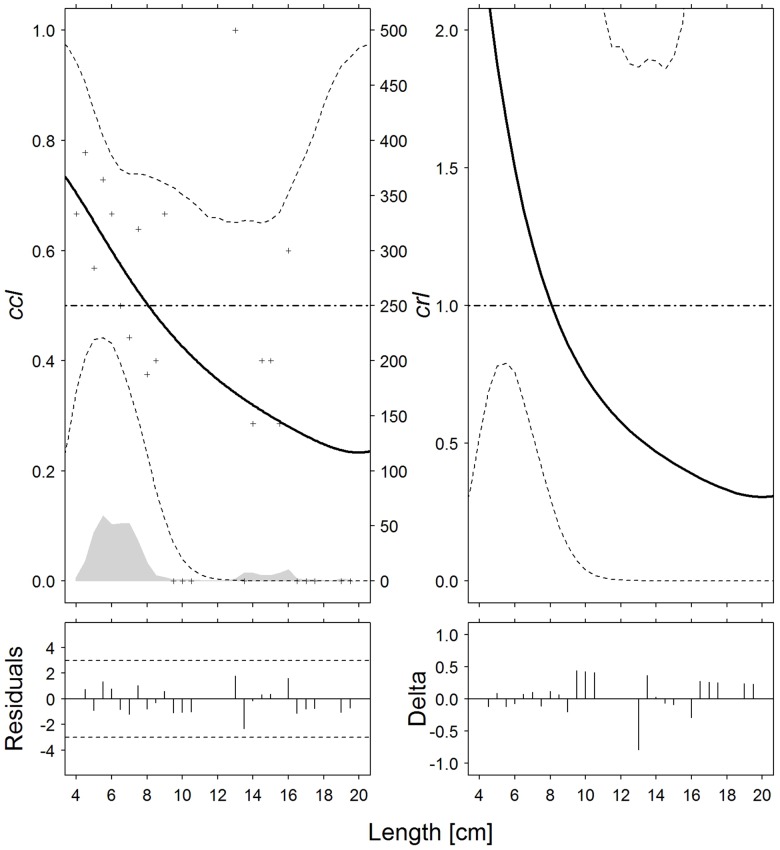
Poor cod (*T*. *minutus*). Catch comparison rates (*cc*_*ℓ*_, *top left*) and catch ratio curves (*cr*_*ℓ*_, *top right*) of the 30-minute and 60-minute tows (solid curves). Crosses: experimental rates; thin dotted curves: 95% confidence intervals; grey solid curve: summed and raised catch populations for all hauls; horizontal dashed line: baseline of no effect on catch performance after changing tow duration. Partial residual plots (*bottom left*) and delta dispersion (*bottom right*) for the two models.

The length-dependent catch comparison rate, *cc*_*ℓ*_, was then estimated and plotted (Figs 1–5, left). The *p*-values obtained for the model fits for the five species were all *>>0*.*05* (*p = 0*.*148–0*.*299*), demonstrating that the experimental data were appropriately described by the models. Low *p*-values (*p = 0*.*055–0*.*074*) were found only for Norway lobster and Atlantic horse mackerel, but given the lack of systematic patterns in the residuals between experimental points and model curves (Figs 3 and 4), this can be considered as a case of overdispersion in the data. These results were therefore used for a further assessment (i.e. the comparison of the catch efficiency of the two tow durations for each one of the five studied species based on the combined catch rate model). The quantitative difference by length class in catch efficiency by size between *T30* and *T60* tows is demonstrated by the catch ratio curves, *cr*_*ℓ*_ (Figs 1–5, right). Because of the mathematical relation between *cc*_*ℓ*_ and *cr*_*ℓ*_, see Eq (10), in the *cr* curve the same length class span, where the CI is < 0.5 in the *cc*_*ℓ*_, is below the baseline 1.0 (and is thus significantly different).

For European hake, curves *cc* and *cr* (Fig 1) indicate that *T30* tows caught fewer individuals than *T60* tows in the 13–32 cm length classes, since *cc*_*ℓ*_ and *cr*_*ℓ*_ were respectively < 0.5 and < 1.0; moreover, given that *cc*_*ℓ*_ = 0.5 and *cr*_*ℓ*_ = 1 are inside the 95% CI for all length classes, they do not show any significant effect of tow duration on catch efficiency for this species. However, the difference appears to be length-dependent, since curves *cc*_*ℓ*_ and *cr*_*ℓ*_ show a monotonic decline, which means that rising body length induces a greater difference in catches between *T30* and *T60* tows.

For surmullet (Fig 2) the pattern was less clear, because the curves first climbed between 9 and 13 cm and then showed a downward trend between 13 and 22 cm. However, considering the confidence limits of the curves, such irregular pattern may be accidental, since curves with different shapes could also fit the experimental data. Notably, for this species the mean curves *cc* and *cr* are also below the horizontal baselines and show a downward trend for length classes > 15 cm.

As regards Nephrops, the shorter tows caught on average a larger number of specimens with a carapace length < 3.3 cm, even though the difference was not significant (Fig 3). As in the case of hake, curves *cc* and *cr* exhibit a constant monotonic decrement; moreover, *T30* tows caught significantly fewer Nephrops > 4.0 cm, because the baselines are not within the confidence limits (Fig 3).

For Atlantic horse mackerel, like surmullet, the catch comparison curves, *cc* and *cr* (Fig 4) show a reverse cup shape. This suggests that in *T30* tows, medium-sized individuals (i.e. 11–16 cm) are more likely caught than small and large ones, contradicting the expectation of a general monotonic progression of the catch comparison curves (i.e. a length dependent effect). The catch comparison curves, *cc* and *cr*, for poor cod are reported in Fig 5. There was no significant effect of tow duration on any of the length classes. However, as in the cases of hake and Norway lobster, the two curves exhibited a monotonous decreasing tendency, with higher proportions of smaller individuals in *T30* tows and lower proportions of larger individuals across the range of specimen size in *T60* tows.

The data reported in Table 3 quantify the relative catch efficiency of the *T30* tows compared with the *T60* in terms of mean catch ratio by size. The results indicate that tow duration does exert an effect on the catch of large fish, even though the difference was significant only for Nephrops. In this species, for carapace lengths > 4.0 cm, the average catch ratios of *T30* tows were significantly below 22% (*p<0*.*001*), whereas the data obtained for small and medium-sized specimens provided no evidence of a consistent effect of tow duration on catch efficiency.

**Table 3:** Estimated values of mean catch ratio *cr*_*ℓ*_ for length class *ℓ*.

Length	HKE	MUX	NEP	HOM	POD
5–7	1.02–1.03	0.32–0.37		0.32–0.46	1.88–1.22
8–11	1.03–1.02	0.43–0.93		0.57–1.03	1.01–0.65
12–14	1.00–0.97	1.11–1.12	3.18–3.42	1.18–1.33	0.58–0.47
15–16	0.95–0.92	0.93–0.66	3.49–3.52	1.26–1.10	0.43–0.39
17–19	0.90–0.85	0.40–0.16	3.52–3.42	0.85–0.35	0.36–0.31
20–21	0.83–0.81	0.11–0.09	3.31–3.18	0.18–0.08	0.30–0.31
22–23	0.79–0.77	0.07–0.06	3.03–2.85	0.03–0.01	0.34–0.39
24–27			2.65–2.02		
28–32			1.81–1.05		
33–39			0.89–0.27		
40–50			0.22–0.01		
**p-value**	0.388	0.299	0.074	0.055	0.148
**DOF**	43	14	21	19	21
**Deviance**	44.99	16.24	31.01	38.28	27.74

Values of p-value, degree of freedom (DOF) and Deviance have been obtained with the combined model for different length classes with 60 minutes tows used as the baseline. The values for *cr*_*avr*_ are calculated based on Eq 7. HKE: European hake (*M*. *merluccius*); MUX: Surmullets (*Mullus* spp); NEP: Norway lobster (*N*. *norvegicus*); HOM: Atlantic horse mackerel (*T*. *trachurus*); POD: Poor cod (*T*. *minutus*). Length classes expressed as [mm] for NEP and as [cm] for the other species.

## Discussion

The goal of this work was to establish whether reducing trawl survey tow time would obviate sub-sampling, increase the number of stations, and improve the accuracy of survey estimates without reducing *CPUE*. The study provided some expected and some unexpected findings. This noted, some caveats are in order. In fact, the present results are based on only fourteen hauls (seven 30 min and seven 60 min tows) and on a limited number of length measurements (see S1 Table). This leaves some uncertainty as to the estimated catch comparison curves. However, since the uncertainties are reflected in the confidence bands around the catch comparison rate and ratio curves and the parameters that are provided with the results, the limited number of fish caught and measured in the study should not be a major concern if the confidence bands are considered when drawing the conclusions.

Some of the results regarding species richness were clearly expected, since increased fishing time obviously increased the probability to catch species. An unexpectedly finding was that the shorter tows caught about 26 species per haul in 30 minutes, and that only around one more species was caught in the next 30 minutes. Nevertheless, tow duration did affect the *CPUE*, as demonstrated by the fact that the total catch or *CPUE* of the main G1 target species in the shorter tows was 60% smaller.

Since a similar difference was also found for G2 species and the overall catch, this finding is not accidental. Moreover, analysis of the data by species demonstrated that the difference often exceeded 50%. These data suggest that, after achieving optimum opening, the gear probably needs additional time to reach full catch efficiency. These results are in line with Goddard [28], who showed a significant *CPUE* decrease for two flatfish species with decreasing tow duration, but contrast with those of Godø et al. [3], who reported that in Norwegian waters short tows were at least as efficient as long tows for cod (*Gadus morhua*), haddock (*Melanogrammus aeglefinus*), and long rough dab (*Hippoglossoides platessoides*). In fact, in their study short tows provided a higher catch per minute than long tows, even though the difference was not significant. Differences existing between the two sets of experiments can at least partly explain the different results. First of all, the 5–6°C of temperature of the Norwegian waters involve different fish reactions compared with the 12°C of the present experiments; moreover, the two studies involve different target species and sizes.

According to most widely shared theory, fish fall back into the trawl due to fatigue after swimming at the trawl speed in the mouth of the net, a mechanism that has been called “catching by exhaustion” [29]. Large fish have a greater swimming capacity than small fish and can swim in front of the trawl for longer periods, whereas small fish swim only briefly in the mouth of the net and show a higher rate of turnover [29, 30]. As a result, short tows would be expected to underestimate the proportion of larger fish in a population. Despite the limited number of hauls, the present study confirmed the general theory and produced consistent results for different species.

Indeed, it indicated that the short tows were less efficient in catching large hake, surmullet, Atlantic horse mackerel, poor cod, and Nephrops, although the difference with the longer tows was significant only for the latter species. The study findings are not in line with some earlier investigations [3–5], which reported that the mechanism responsible for changes in *CPUE* with tow duration operates uniformly over the size ranges of the species examined. Further works is clearly needed to gain insight into the question.

The lack of size selection by tow duration has tentatively been explained by Godø et al. [3] with the surprise factor, whereby schooling in front of the trawl would induce an alert reaction at an earlier stage in the catching process, thus reducing the probability of capture. Accordingly, “catching by surprise” would be most effective in the first few minutes of a tow, before school formation. While the exhaustion effect depends on swimming capacity and specimen size, the surprise effect does not appear to be related to size. Although Somerton et al. [4] and Walsh [5] have found an increase in *CPUE* with decreasing tow time, they have rejected the “catch by surprise” hypothesis, because the low water temperature during their experiment would have reduced swimming capacity to such an extent that the herded fish would have been too few to elicit an escape reaction from other fish.

## Conclusions

Tow duration affected *CPUE*, trawl catch performance, and the rates of the main species sampled. *CPUE* decreased in the shorter tows for 11/12 species sampled, but the difference was significant only for Nephrops. The shorter tows were less efficient than the longer tows in catching large specimens. Because the catching rate of some species was size-dependent, the size frequency distributions obtained from the short tows may misrepresent the actual size frequency distributions of populations in the area, as in the case of all multispecies trawl studies. Since a major goal of standardised trawl surveys is to provide data series to estimate trends in biological parameters such as abundance indices and size distributions, the need for supporting series consistency could met by leaving surveys in their “imperfect state” [31], even though the approach may seem to accept inefficiency. Assessment of the escapement behaviour of large individuals of some species, which was not detected in the present experiments, requires further work.

## Future works

Future work is expected to provide further insight into the mechanisms underlying the effect of tow duration. Another experiment carried out in deep waters, where *T60* are conducted during MEDITS program, and with more hauls and measured individuals should be done in the future. Finding a way to reduce tow duration without reducing *CPUE* would obviate sub-sampling and allow increasing the total number of stations, thus improving the accuracy of survey estimates.

## Supporting information

S1 File Design of the GOC 73 trawl, the standard trawl designed for the MEDITS programme by IFREMER Sète. Its main characteristics are: headline 35.7 m, sidelines 7.4 m, footrope 40.0 m, two panels with sides, for a boat of 500–1000 HP, pull at bollard 4.5 t, twine area 54.78 m. PA = polyamide, PE = polyethylene, PP = polypropylene, SST = stainless steel, ST = steel. The mesh number of the netting panel width does not include selvedge meshes. Five meshes (6 knots) per selvedge should be added where indicated. Conversely, to obtain panel depth, a row (1/2 mesh) should be subtracted from each panel, since the joining row is included in the mesh count.
(DOCX)

S1 Table Conditions of the fishing experiments conducted with the two types of tows, which had a nominal duration of 30 or 60 minutes (*T30* and *T60*, respectively). Mean effective tow duration, towing speed (TS), horizontal net opening (HNO), swept longitudinal distance (SLD), swept area (SWA), and total catch data are provided. The *kratio* is the ratio of SWA to its maximum value (Max) attained in the hauls. For each species is reported the total number of measured individuals. HKE: European hake (*M*. *merluccius*); MUX: Surmullets (*Mullus spp*); NEP: Norway lobster (*N*. *norvegicus*); HOM: Atlantic horse mackerel (*T*. *trachurus*); POD: Poor cod (*T*. *minutus*).
(DOCX)
