# Supplementary figures and images for: Influence of tow duration on catch performance of trawl survey in the Mediterranean Sea
Source: PLoS One. 2018 Jan 22;13(1):e0191662. doi: 10.1371/journal.pone.0191662 (PMC5777655; doi:10.1371/journal.pone.0191662)

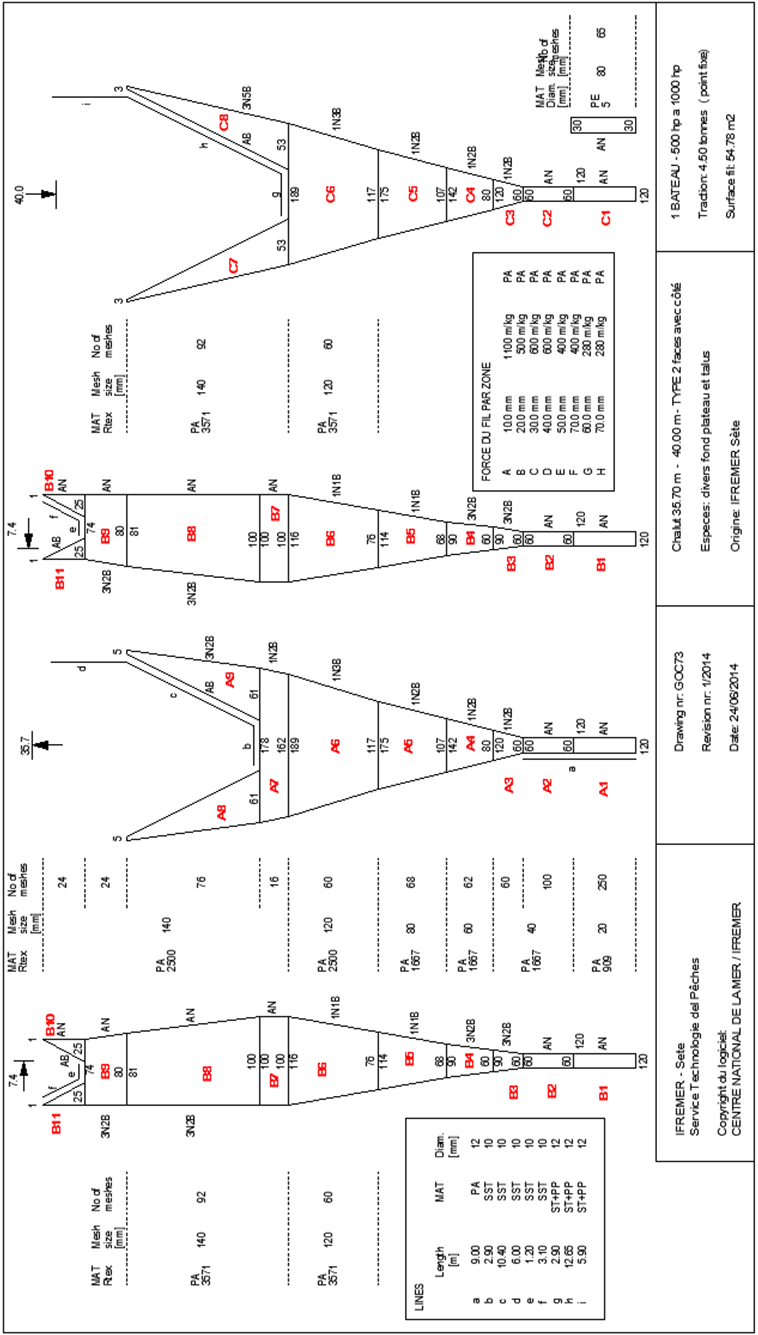

Supplement: S1 File — Its main characteristics are: headline 35.7 m, sidelines 7.4 m, footrope 40.0 m, two panels with sides, for a boat of 500–1000 HP, pull at bollard 4.5 t, twine area 54.78 m. PA = polyamide, PE = polyethylene, PP = polypropylene, SST = stainless steel, ST = steel. The mesh number of the netting panel width does not include selvedge meshes. Five meshes (6 knots) per selvedge should be added where indicated. Conversely, to obtain panel depth, a row (1/2 mesh) should be subtracted from each panel, since the joining row is included in the mesh count. (DOCX) [file pone.0191662.s001.docx]
