# Supplementary material for: Influence of tow duration on catch performance of trawl survey in the Mediterranean Sea
Source: PLoS One. 2018 Jan 22;13(1):e0191662. doi: 10.1371/journal.pone.0191662 (PMC5777655; doi:10.1371/journal.pone.0191662)
Supplement: S1 Table — Mean effective tow duration, towing speed (TS), horizontal net opening (HNO), swept longitudinal distance (SLD), swept area (SWA), and total catch data are provided. The kratio is the ratio of SWA to its maximum value (Max) attained in the hauls. For each species is reported the total number of measured individuals. HKE: European hake (M. merluccius); MUX: Surmullets (Mullus spp); NEP: Norway lobster (N. norvegicus); HOM: Atlantic horse mackerel (T. trachurus); POD: Poor cod (T. minutus). (DOCX) [file pone.0191662.s002.docx]

S1 Table. Conditions of the fishing experiments conducted with the two types of tows, which had a nominal duration of 30 or 60 minutes (*T30* and *T60*, respectively).

| **Haul** | **Type** | **TT** | **TS** | **HNO** | **LDE** | **SWA** | **Catch** | | ***kratio*** |  | **HKE** | **MUX** | **NEP** | **HOM** | **POD** |
| --- | --- | --- | --- | --- | --- | --- | --- | --- | --- | --- | --- | --- | --- | --- | --- |
|  |  | [min] | [kn] | [m] | [m] | [km^2^] | [kg] | [kg/km^2^] | [-] |  |  |  |  |  |  |
| 919 | T60 | 59 | 3.17 | 15.43 | 5784 | 0.089 | 18.73 | 209.87 | 0.955 |  | 55 | 5 | 6 | 21 | 20 |
| 921 | T60 | 60 | 3.09 | 13.76 | 5718 | 0.079 | 14.64 | 186.08 | 0.842 |  | 71 | 6 | 19 | 17 | 22 |
| 923 | T60 | 60 | 3.08 | 16.21 | 5699 | 0.092 | 9.87 | 106.79 | 0.989 |  | 27 | 1 | 18 | 299 | 21 |
| 925 | T60 | 60 | 2.96 | 15.63 | 5432 | 0.085 | 9.40 | 110.75 | 0.909 |  | 32 | 4 | 29 | 131 | 4 |
| 927 | T60 | 57 | 3.25 | 16.45 | 5680 | 0.093 | 16.70 | 178.76 | 1.000 |  | 32 | 1 | 13 | 404 | 15 |
| 930 | T60 | 60 | 3.10 | 13.79 | 5704 | 0.079 | 11.68 | 148.52 | 0.842 |  | 40 | 8 | 9 | 11 | 46 |
| 932 | T60 | 58 | 3.25 | 15.53 | 5780 | 0.090 | 14.28 | 159.08 | 0.961 |  | 49 | 6 | 7 | 33 | 16 |
| 920 | T30 | 30 | 3.02 | 14.87 | 2748 | 0.041 | 5.08 | 124.32 | 0.437 |  | 26 | 1 | 3 | 6 | 5 |
| 922 | T30 | 32 | 3.32 | 14.76 | 3276 | 0.048 | 8.36 | 172.90 | 0.518 |  | 35 | 5 | 2 | 5 | 31 |
| 924 | T30 | 31 | 3.13 | 17.00 | 3025 | 0.051 | 4.03 | 78.32 | 0.550 |  | 14 | 1 | 13 | 119 | 10 |
| 926 | T30 | 30 | 3.06 | 15.58 | 2784 | 0.043 | 2.57 | 59.25 | 0.464 |  | 12 | 0 | 9 | 38 | 8 |
| 928 | T30 | 30 | 3.23 | 16.43 | 2939 | 0.048 | 4.21 | 87.16 | 0.517 |  | 16 | 0 | 10 | 212 | 17 |
| 929 | T30 | 40 | 2.96 | 14.99 | 3636 | 0.055 | 6.30 | 115.60 | 0.583 |  | 30 | 6 | 1 | 10 | 21 |
| 931 | T30 | 31 | 3.09 | 14.82 | 2970 | 0.044 | 4.60 | 104.42 | 0.471 |  | 12 | 1 | 2 | 3 | 9 |
|  | **Max** | 60 | 3.32 | 17.00 | 5784 | 0.093 |  |  | **T60** |  | 306 | 31 | 101 | 916 | 144 |
|  |  |  |  |  |  |  |  |  | **T30** |  | 145 | 14 | 40 | 393 | 101 |

Mean effective tow duration (TT), towing speed (TS), horizontal net opening (HNO), longitudinal distance explored (LDE), swept area (SWA), and total catch data are provided. The *kratio* is the ratio of SWA to its maximum value (Max) attained in the hauls. For each species is reported the total number of measured individuals. HKE: European hake (*M. merluccius*); MUX: Surmullets (*Mullus spp*); NEP: Norway lobster (*N. norvegicus*); HOM: Atlantic horse mackerel (*T. trachurus*); POD: Poor cod (*T. minutus*).
